# Beyond reconstruction: minimally invasive aesthetic and regenerative medicine in postoperative oral and maxillofacial surgery

**DOI:** 10.1007/s10006-026-01651-4

**Published:** 2026-10-13

**Authors:** Michael Alfertshofer, Alexander G. M. Hopf, Kilian Kreutzer, Carolin Bestendonk, Christian Doll, Sebastian Cotofana, Norbert Neckel, Max Heiland, Marietta Hopf

**Affiliations:** 1 Department of Oral and Maxillofacial Surgery, Charité – Universitätsmedizin Berlin, corporate member of Freie Universität Berlin, Humboldt-Universität zu Berlin, Berlin, Germany; 2 Department of Plastic Surgery, Technical University of Munich, Munich, Germany; 3 Institute of Psychology, University of Innsbruck, Innsbruck, Austria; 4 Department of Plastic and Reconstructive Surgery, Guangdong Second Provincial General Hospital, Guangzhou, Guangdong China; 5 Department of Dermatology, Erasmus Medical Centre, Rotterdam, The Netherlands; 6 Centre for Cutaneous Research, Blizard Institute, Queen Mary University of London, London, UK; 7 Department of Plastic Surgery, Vanderbilt University Medical Center, Nashville, TN USA

**Keywords:** Aesthetic medicine, Regenerative medicine, Oral and maxillofacial surgery, Polynucleotides, Hyaluronic acid, Botulinum toxin

## Abstract

**Purpose:**

Oral and maxillofacial reconstruction restores anatomical continuity and function after tumor resection, trauma and congenital deformity, yet patients frequently remain affected by fibrosis, scar tethering, impaired oral competence, asymmetry and altered dynamic facial function. These deficits can impair quality of life, social interaction and body image. Conventional revision surgery carries additional morbidity, and minimally invasive options have emerged as a postoperative adjunct. This article reviews how minimally invasive aesthetic and regenerative medicine can extend reconstructive rehabilitation in oral and maxillofacial surgery.

**Methods:**

Current biological and clinical evidence for polynucleotides (PN), hyaluronic acid (HA) and botulinum toxin A (BoNT-A) was reviewed and integrated with selected, non-consecutive illustrative cases from a dedicated aesthetic and regenerative consultation in an academic oral and maxillofacial surgery department. Clinical applications were illustrated across four patient categories: oncological, congenital, trauma/scar and aesthetic-functional sequelae after surgery.

**Results:**

In illustrative cases from different categories, a staged approach of tissue conditioning, structural restoration and dynamic neuromodulation was associated with clinically observed and patient-reported improvements in scar pliability, tissue gliding, oral competence, dynamic symmetry and quality of life. PN were used primarily for regenerative tissue conditioning, HA for reversible structural restoration, and BoNT-A to modulate muscular imbalance and pathological wound tension. As interventions were frequently combined and no validated outcome measures or controls were used, these observations are hypothesis-generating.

**Conclusion:**

Minimally invasive regenerative and aesthetic treatment may serve as a functional extension of reconstruction rather than a cosmetic adjunct. The central concept is not volumisation, but restoration of tissue quality, dynamic balance and facial function. Prospective studies using validated functional, scar and patient-reported outcome measures are required to define its role in modern oral and maxillofacial surgery.

## Introduction

Reconstructive oral and maxillofacial surgery is governed by the imperative of restoring anatomical continuity. After ablative tumor resection, high-energy trauma or the staged repair of a congenital malformation, the functional closure of the defect is achieved reliably. The patient, however, lives in what remains once this anatomical continuity has been secured. In this context, local and free flaps are dependable but rarely match the colour, pliability, mobility and dynamic behaviour of the tissue they ought to replace [1–3]; irradiated skin becomes atrophic, fibrotic and poorly perfused [4]; and scars contract along functionally critical lines such as the lip, the oral commissure and the lower eyelid [5, 6], potentially converting an anatomically successful reconstruction into an aesthetic, functional and psychosocial problem.

The psychosocial weight of this residual burden is well documented. Facial disfigurement is consistently described as one of the most challenging consequences of head and neck cancer treatment: In a series of three cross-sectional studies, Fingeret et al. mapped the trajectory of body-image concerns throughout the treatment course of head and neck cancer patients. Before surgery, concerns were generally low, while after treatment, however, the burden rose sharply: in a surgical cohort of 280 patients at risk of facial disfigurement related to a surgical intervention, 75% reported body-image concerns at some point since diagnosis. A longitudinal analysis confirmed that concerns increased significantly post-operatively and remained elevated beyond one year. Crucially, distress was greatest in patients with functional deficits of speech and swallowing rather than appearance alone, and across the series higher body-image dissatisfaction consistently tracked with poorer quality of life [7–9]. Subsequent work by Macias et al. has shown that up to almost one third of survivors meet the criteria for clinically significant body-image-related distress (BID), a state these authors link to depression, social isolation and reduced quality of life [10]. This distress is not merely a quality-of-life concern but may carry prognostic weight, as depression arising after a head and neck cancer diagnosis has further been associated with worse survival, an effect shown to be particularly pronounced in female patients [11]. 

The face is central to personal identity and social interaction. Therefore, even deficits that are anatomically minor can be functionally and emotionally major; it is precisely this evidence base that justifies framing the residual deficits addressed here as a rehabilitative rather than a mere “cosmetic problem”. Beyond identity and appearance, the face is the primary organ of interpersonal communication [12]. Consequently, postoperative deficits affecting facial mobility, symmetry, tissue pliability or oral competence may alter not only appearance, but also emotional expression and social interaction [11, 13–16]. 

Oftentimes, such residual deficits occupy a middle ground and highlight a common dilemma in surgical aftercare of these patients: They are usually too minor to justify a further revision surgery, yet too disabling to ignore. An incompetent oral seal causes drooling, difficulty drinking, articulation problems and social withdrawal [17]; a tethered philtrum or an asymmetric vermilion after cleft repair is a lifelong marker [18]; a contracted lower-eyelid scar after periocular tumor excision produces reflex epiphora and a chronically irritable eye [19]. For this final stage of rehabilitation, conventional surgery offers comparatively blunt instruments.

On the other hand, minimally invasive injectables do not require anesthesia – neither general and in most of the cases nor local -, are office-based, repeatable and, in the case of the volumizing agents such as HA, also reversible [20]. PN and polydeoxyribonucleotides (PDRN) act through the adenosine A2A receptor and the nucleotide salvage pathway to modulate inflammation, support fibroblast activity and promote angiogenesis [21]; BoNT-A reduces wound tension and modulates fibroblast behaviour, with randomized evidence for improved scarring [22] and for functional rebalancing in facial palsy [23]; and cross-linked HA restores small, defined volumes [24]. Applied in a multimodal and mechanism-led manner, these minimally invasive modalities may address the residual deficits of the postsurgical face.

To our knowledge, no previous overview has integrated these modalities into a single mechanism-led pathway organized by surgical patient category, which is the gap this article sets out to address. On this premise our department established a dedicated aesthetic and regenerative consultation for surgically treated patients to connect both worlds: surgical-driven restoration of anatomical continuity with minimally invasive-driven rehabilitation of function and aesthetic appearance to optimize the final result.

## The aesthetic and regenerative consultation

### Concept, substances and treatment principles

The consultation is offered to patients of the oral and maxillofacial surgery department who have completed their reconstructive pathway and retain a functional or aesthetic deficit. The aim of the consultation is to improve function, overall aesthetic appearance, tissue quality, symmetry, facial expression and ultimately quality of life. Every patient undergoes a structured analysis before any intervention, documenting the functional problem, for instance oral competence (e.g., insufficient lip closure with saliva loss), scar-related contractures (e.g., insufficient mouth opening), altered lid position (e.g., ectropion), fibrosis (e.g., after radiation therapy), atrophy and volume loss of transplanted tissue, and the pattern of asymmetry at rest and in animation [25]. Treatment follows a staged multimodal approach consisting of three sequential principles: tissue regeneration and scar conditioning, conservative structural restoration, and finally modulation of dynamic movement. The first stage focuses predominantly on improving tissue quality, reducing fibrosis and releasing scar tethering, most commonly through PN-based regenerative treatment [26, 27]. Once tissue quality has improved and reached better pliability, selective and reversible structural restoration is performed where required, primarily using HA with the specific rheological properties adapted to the biomechanical demands of the affected region [28]. The final stage addresses dynamic imbalance and irregular muscular pull through targeted BoNT-A treatment. Therefore, we are aiming for a staged minimally invasive process rather than a single intervention, addressing tissue quality, structural deficits, contour and dynamic movement, with differing extents depending on each individual patient case. In practice, the stages were not always separated in time. In some of the illustrative cases, subcision, PN and HA were combined within one session (Table 2). The proposed sequence is therefore a biologically motivated framework derived from clinical experience instead of a validated protocol based on currently available scientific evidence.

### Polynucleotides (PN)

PN are highly purified DNA fragments most commonly of salmon origin, that are naturally degraded in the tissue to nucleosides that act through the adenosine A2A receptor and the nucleotide salvage pathway. PN and PDRN share the origin and mechanism but differ in fragment length and molecular weight, with PDRN consisting of shorter, lower-molecular-weight fragments [29, 30]. This pharmacological rationale is effectively illustrated by the experimental work of Galeano et al. and Jeong et al. [29, 31] The principal downstream effects are threefold: (i) an anti-inflammatory action, with A2A-receptor stimulation suppressing nuclear factor-κB (NF-κB) signaling and the associated reduction in interleukin-6 (IL-6) and tumour necrosis factor-α (TNF-α), an effect demonstrated directly in human gingival fibroblasts and oral mucosal epithelial cells by Picciolo et al. [32]; (ii) stimulation of fibroblast proliferation and extracellular-matrix deposition and reorganisation [33, 34]; and (iii) a pro-angiogenic effect mediated in part through upregulation of vascular endothelial growth factor (VEGF) [29, 35]. PN further exhibit a favourable safety profile, in keeping with their physiological mechanism by supplying deoxyribonucleotides in a non-particulate fashion. Common adverse events seen with PN are therefore limited to mild, transient local reactions and no reported granuloma formation or vascular occlusion [30]. The clinical scar literature is now growing: Kim et al. showed in a randomized double-blinded controlled trial after thyroidectomy that PN injections produced better scar quality with significantly lower Vancouver Scar Scale scores than controls, a finding echoed for PDRN in the early-postoperative randomized trial of Kim et al. in the same setting [36, 37]. Likewise, Araco and Araco demonstrated in a prospective randomized placebo-controlled study that PN treatment was safe and effective in moderate to severe atrophic acne scars [38]. Specifically for the oral cavity, the 2025 systematic review by Mari et al. concluded that PDRN injections are capable of improving periodontal regeneration with consistent gains in clinical attachment, bone fill and probing-depth reduction, building on previous experimental periodontitis and mucositis work [39, 40]. The resulting clinical rationale is that PN act on the dermal fibroblasts and prompt the tissue to rebuild its own collagen, elastin and HA, thereby setting the foundation to improve several aspects of tissue quality at once, including hydration, regeneration, and antioxidant and anti-inflammatory tone, so that they are intended to condition tissue rather than to fill it. These data derive largely from experimental models and from periodontal, mucosal, acne-scar and thyroidectomy settings; their transfer to irradiated, post-oncological maxillofacial tissue has not been studied and represents biological rationale rather than established evidence.

### Hyaluronic acid (HA)

HA primarily serves as a reversible tool for structural restoration and tissue support, while also contributing to hydration, tissue integration and biomechanical tissue quality. Its clinical value depends on selecting the appropriate rheology for the tissue: firmer, highly cross-linked gels may provide deep support and projection, whereas softer formulations are better suited for subtle contour harmonization in mobile or more delicate postsurgical areas [28, 41, 42]. Depending on the degree of cross-linking, tissue integration and injection plane, treatment durability may range from several months to, in selected structural indications, up to approximately 18–24 months with a single treatment [43]. An important safety feature of HA injections is the enzymatic reversibility with hyaluronidase, which can act within minutes to hours to dissolve the product, e.g. in case of a vascular occlusion [20]. Yet, mechanistic reversibility does not guarantee recovery: filler-related visual loss frequently persists despite prompt hyaluronidase treatment [44, 45]. Its reconstructive use after trauma, tumor resection and congenital deformity is reported mostly as small series and case reports rather than controlled trials; Schweiger et al. described the first use in a repaired cleft lip [46], and Rivers et al. [47] and Thomson et al. [48] have since reported structured adult-cleft experience with patient-reported outcomes, while periocular use in particular requires caution because vascular and compressive complications are well described. In addition to its structural properties, HA exhibits biological activity within the extracellular matrix, partly mediated through CD44 receptor interactions, thereby having been reported to influence hydration, fibroblast activity and tissue homeostasis [49]. This combination of structural and biological activity underpins the value of HA within a staged, regenerative treatment protocol.

### Botulinum toxin A (BoNT-A)

BoNT-A is an effective neuromodulator that reversibly reduces muscular activity through cleavage of SNAP-25 and subsequent inhibition of acetylcholine release at the neuromuscular junction [50]. Depending on the formulation, dose and target muscle, its clinical effects typically persist for several months. In the reconstructive setting, this allows targeted modulation of muscular imbalance, dynamic asymmetry and excessive wound tension with minimal invasiveness and high anatomical precision [51]. Following the original prospective blinded placebo-controlled trial of Gassner et al. [22], subsequent split-scar randomized controlled trials by Hu et al. [52] and Kim et al. [53], together with meta-analyses pooling more than five hundred wounds, have shown that peri-incisional injection can improve scar appearance and width by reducing wound tension and modulating fibroblast differentiation [54]. Previous literature further focused on functional rehabilitation and how BoNT-A can improve symmetry, synkinesis and quality of life in long-standing facial palsy, supporting its use to rebalance dynamic asymmetry after reconstruction [55–57]. 

### Other regenerative modalities

Beyond injectables, a growing range of regenerative and energy-based modalities can further support biologically guided postoperative rehabilitation. The choice among them is made case by case, matched to the individual patient’s tissue characteristics, deficit pattern, and rehabilitative goal. Autologous platelet concentrates (i.e., PRP, PRF) offer similar regenerative intent but a shorter-lived stimulus than PN, and because they release TGF-β they may reinforce fibrosis: a limitation in already-scarred tissue [58]. Energy-based approaches such as radiofrequency (including microneedling RF) can drive dermal remodelling and tightening [59]. Biostimulatory agents such as poly-L-lactic acid (PLLA) and calcium hydroxylapatite (CaHA) may support collagen remodelling in patients with generalized atrophy or ageing-related decline, though careful attention to injection depth and distribution is needed to avoid nodule formation [60, 61]. These adjuncts remain supportive rather than central in our current practice.

### Assessment and documentation

This article presents a narrative, clinically illustrative overview of a structured multimodal treatment concept rather than a formal hypothesis-testing study. Patients are documented using photographs at baseline, after each session and at follow-up, and are clinically assessed for the respective aesthetic or functional concern, complemented by structured patient-reported feedback. No validated functional, scar or patient-reported outcome instruments, independent raters or control groups were used, and outcomes were not quantified. Treatment is performed in an outpatient setting under no or local anaesthesia, with sessions typically repeated at intervals of three to six weeks, depending on tissue response and treatment goals. Care is taken with patient selection: where the history of any previous injectable treatment cannot be reliably established, further intervention is avoided. In oncological patients, treatment has been considered only after completion of oncological therapy, without clinical or radiological evidence of recurrence at the latest follow-up. Further, treatments were only performed in the absence of active infection, wound dehiscence, osteoradionecrosis or exposed osteosynthesis material in the treatment area. The cases presented are intended to illustrate the spectrum of indications and underlying treatment principles rather than a consecutive cohort and were selected by the authors to represent each of the four patient categories, based on the availability of complete photographic documentation and written consent for publication. Treatments, follow-up, outcomes and adverse events are summarized in Table 2. All products utilized in the cases presented herein were manufactured by Croma-Pharma, Leobendorf, Austria. Since subcision, PN and HA were frequently combined, observed improvements cannot be attributed to an individual agent, including PN, or to the treatment sequence itself. Prospective evaluation using validated functional, scar and patient-reported outcome measures is therefore an important next step for the field.

## Clinical applications across patient categories

### Spectrum of patients and indications

Although tumor, congenital, traumatic and aesthetic patients differ in etiology and surgical history, they converge on a shared pattern of compromised tissue quality, structural imbalance and disturbed dynamic muscle function, which is best understood across four clinical categories, each with distinct deficits, biological challenges and therapeutic priorities (Table 1).

**Table 1:** Patient categories after maxillofacial surgery, typical residual deficits and what minimally invasive aesthetic and regenerative treatment may add

Patient category	Typical residual deficit	What minimally invasive treatment may add
**Oncological patients**	Radiation fibrosis, hypovascular and tethered flap bed, incompetent oral seal, drooling, volume loss	PN conditioning, scar release, restrained reversible volume, dynamic rebalancing
**Congenital malformations**	Philtral scar, vermilion notch, Cupid bow asymmetry, upper-lip volume deficit, lip incompetence	PN scar conditioning then hyaluronic acid contouring; tension-reducing botulinum toxin at scar revision
**Trauma and scars**	Contracted, adherent or hyperdynamic scars, mimetic imbalance, restricted mobility	Peri-incisional botulinum toxin, subcision and PN, selective hyaluronic acid
**Aesthetic patients**	Periocular lid malposition and epiphora, contour over implants and bony defects, post-oncologic appearance burden	Botulinum toxin for lid dynamics and symmetry, restrained reversible contouring

### Oncological patients

**Fig. 1:**
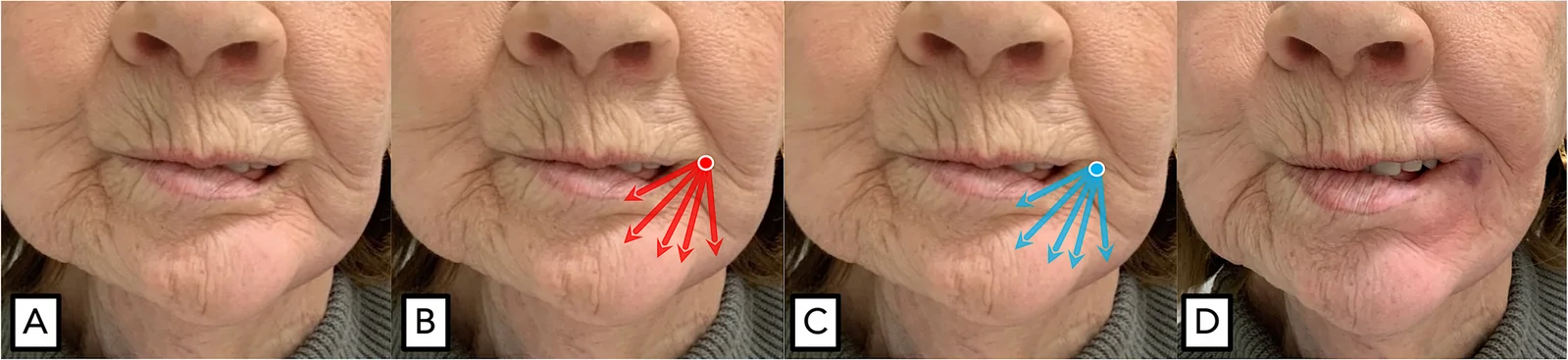
Multimodal treatment of lower lip dysfunction in a 75-year-old female patient following lower lip reconstruction after squamous cell carcinoma of the lower lip. The patient presented with impaired lip competence, oral leakage during eating and drinking, and reduced perioral mobility. (**A**) Baseline appearance demonstrating lower lip asymmetry, soft tissue deficiency, and compromised oral closure. (**B**) Initial regenerative treatment with 2 ml of PolyPhil (red): after instillation of 0.3 ml local anaesthetic (without adrenaline) at the cannula entry point and perforation with a sharp needle, a blunt cannula was advanced in tunnelling technique to deliver a further 1–2 ml of local anaesthetic to the treatment area, followed by fan-shaped retrograde linear threading of the PN through the same single entry point to improve tissue quality and soft tissue flexibility. (**C**) Subsequent structural restoration using 1 ml of medium cross-linked Saypha Filler (blue), delivered through the same entry point to provide support, improve lower lip contour, and enhance functional lip competence. (**D**) Immediate post-treatment result after one session demonstrating improved lower lip support, enhanced oral closure

The oncological patient is the paradigmatic candidate for an aesthetic and regenerative add-on. Resection and flap reconstruction, frequently combined with radiotherapy, leave a bed that is fibrotic, hypovascular and poorly elastic, with intra-oral and cutaneous scar tethering, volume loss and, very often, an incompetent oral closure with salivary loss and restricted perioral mobility [4, 5, 17]. The rationale for a regenerative-first approach in this group is biologically plausible. The biology of the irradiated, chronically inflamed scar is the target that PN are intended to modulate [26, 27, 32]. Evidence that PDRN improve periodontal regeneration, demonstrated in the systematic review of Mari et al. [40], and reduce radiation-induced oral mucositis, reported by Podlesko et al. [39], may be relevant to the intra-oral and perioral problems of these patients, although these data derive from non-irradiated periodontal tissue and from topical application in mucositis, respectively, and cannot be directly transferred to irradiated, post-oncological tissue. The principles central to the aesthetic and regenerative treatment of oncological patients are exemplified in a 75-year-old woman after lower-lip reconstruction for a squamous cell carcinoma of the lower lip. She presented with incompetent oral seal, salivary spillage and restricted perioral movement on a fragile, fibrotic bed with intra-oral scar contracture, reduced elasticity, multiple asymmetries, reduced perfusion and volume loss. Treatment was deliberately regeneration-led, namely cautious mechanical release through subcision with a 22G 38 mm cannula combined with PN (2 ml Polyphil), followed by a restrained build-up of volume with HA (1 ml Saypha Filler). A 22G rather than the aesthetic-standard 25G cannula is used in these fibrotic beds to release adhesions as mechanically effectively as possible. Clinically, the patient reported markedly improved drinking function and near-complete restoration of oral seal before the second treatment session. As in most patients of this group, several sessions were needed for a durable result, but with low downtime, no additional operation and a short treatment time of approximately 20 to 30 min. (Fig. 1; Table 2)

**Table 2:** Illustrative cases: treatment per session, follow-up, outcomes and adverse events

Case (Figure)	Patient, category, history	Treatment per session	Follow-up	Outcome (assessment)	Adverse events
1 (Fig. 1)	75-year-old woman; lower-lip reconstruction after squamous cell carcinoma of the lower lip; six prior surgeries and radiotherapy.	S1: Cannula subcision of scar adhesions in the lower lip and chin, followed by fan-shaped injection of 2 mL polynucleotides (PolyPhil) and 1 mL hyaluronic acid (Saypha Filler) through a single entry point using a 22G × 38 mm blunt cannula. S2: Further treatment with Saypha Volume and polynucleotides was planned.	Clinical assessment 4 weeks after S1, before S2.	After S1, drinking function had markedly improved and oral seal was near-complete (patient report and clinical assessment). Figure 1D shows the immediate result after S1.	Transient bruising lasting approximately 1 week; no other adverse events reported.
2 (Fig. 2)	76 y, female; oncological SCC floor of mouth/alveolar ridge, flap reconstruction, RT 4 years earlier	S1: 2 mL polynucleotides (PolyPhil) and 1 mL hyaluronic acid (saypha^®^ Volume) were administered in a fan-shaped pattern using a blunt cannula through a single entry point. S2 (6 weeks after S1): Further cannula subcision through a single entry point using a blunt cannula, with 2 mL polynucleotides (PolyPhil) in the scar tissue.	Clinical assessment 6 weeks after S1 (immediately before S2) and after S2.	At 6 weeks after S1, drinking ability had improved and oral seal was nearly complete but still required active contraction; after S2, mouth closure was possible at rest, with marked improvement in drinking function and quality of life (patient report, clinical assessment).	Transient swelling and a small hematoma; no other adverse events reported.
3 (Fig. 3)	61 y, female; left mandibular osteosarcoma; five operations, fibular free-flap reconstruction and chemotherapy; no radiotherapy. Left perioral and mandibular soft-tissue deficiency with marked asymmetry.	S1 (single session): Cannula subcision of fibrotic adhesions, followed by 2 mL PN (PolyPhil) and 1 mL HA (saypha^®^ Volume Plus) in the marionette region and along the mandibular contour.	Immediately after S1.	Improved contour and symmetry at rest and during movement immediately after treatment (clinical, photographic).	Mild transient erythema and swelling; no other adverse events reported.
4 (Fig. 4)	17 y; congenital Secondary cleft-lip deformity	S1: PN 1 ml (sharp needle) S2: PN 1 ml (sharp needle + needling) S3: Cannula release of residual tethering with right-sided augmentation	Clinical and photographic assessment 4 weeks after S2.	Softening of the philtral scar, improved colour match with the surrounding skin and reduced scar elevation; more symmetric vermilion and lip contour (clinical and photographic assessment). The patient reported high satisfaction.	Transient swelling, mild ecchymosis (~ 1 week); no other adverse events.
5 (Fig. 5)	44 y, male; trauma Bicycle accident, multiple operations, scar revision 2 years earlier	S1: Single entry point; cannula-assisted fan-shaped PN 2 mL (PolyPhil^®^, Croma) to release scar-associated adhesions S2: HA 1 mL (Saypha^®^ Filler, Croma) through the same entry point	Clinical and photographic assessments at 2 and 4 months after treatment.	Reduced scar-related retraction and prominence, softening of scar tissue, improved facial symmetry with smoother perioral and cheek contour at 2 and 4 months (clinical, photographic).	Transient swelling, mild ecchymosis (~ 1 week); no other adverse events.
6 (Fig. 6)	42 y, male; intra-oral scar contracture after genioplasty, lower-lip reduction and intraoral scar revision (6 operations), discomfort and restricted lower-lip movement	S1 (single): needle subcision + PN (PolyPhil^®^) 2 ml (30G, deep and superficial)	Clinical assessment 4 weeks after S1	Resolution of tethering sensation (patient report); improved lower-lip mobility (clinical)	No adverse events were observed or reported
7 (Fig. 7)	81 y, male; aesthetic-functional Lower-lid entropion-type malposition after periocular BCC excision	S1 (single session): Letibotulinumtoxin A (BoNT-A), 4 IU total, injected at four sites on the left side (1 IU per site).	Clinical assessment at 2 weeks and 1 year after S1	Resolution of epiphora at 2 weeks, stable at 1 year without retreatment (clinical, patient report)	No adverse events were observed or reported

Outcomes are based on clinical examination, photographs and patient reports; no validated outcome instruments were used. All sessions were performed under no or local anaesthesia. BCC, basal cell carcinoma; BoNT-A, botulinum toxin A; HA, hyaluronic acid; IU, international units; PN, polynucleotides; RT, radiotherapy; S, session; SCC, squamous cell carcinoma; y, years

**Fig. 2:**
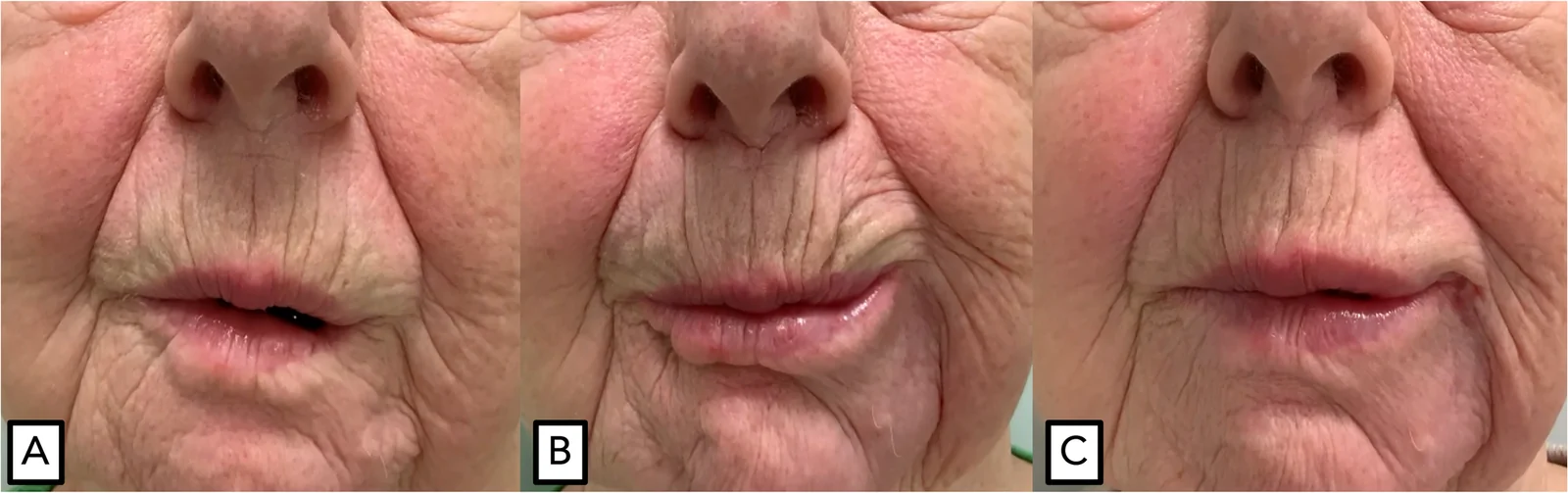
Illustrative treatment course of a 76-year old female patient following resection of a squamous cell carcinoma of the floor of mouth and left alveolar ridge with flap reconstruction and radiotherapy four years earlier, presenting with impaired lip competence and oral leakage due to scar tethering. (**A**) Baseline appearance demonstrating lower lip asymmetry, soft tissue deficiency, and compromised oral closure (**B**) Improved oral competence after the first session of 2 ml of PolyPhil and 1 ml of Saypha Volume injection to the perioral and lower lip area, with competent mouth closure possible under forced muscle contraction (**C**) Further improvement of oral competence after the second session including cannula subcision and PN, with mouth closure now possible at rest and the patient reporting vastly improved drinking function and quality of life

The second case, a 76-year old female patient following resection of a squamous cell carcinoma of the floor of mouth and left alveolar ridge with flap reconstruction and radiotherapy four years earlier demonstrates that regenerative modulation may also address intra-oral scar tethering and functional restriction. She presented with impaired lip competence and oral leakage due to scar tethering, with lower-lip asymmetry, soft tissue deficiency and compromised oral closure. The first session combined PN and HA (2 ml Polyphil and 1 ml Saypha Volume), after which mouth closure was possible only under forced contraction; at the second session a cannula subcision technique was used together with application of PN in the scar tissues, after which closure became possible at rest. Mouth opening and lip competence improved progressively, with marked patient-reported improvement in drinking function and quality of life, illustrating that regenerative work is not confined to the skin surface but may help release the intra-oral tethering that limits function. (Fig. 2)

**Fig. 3:**
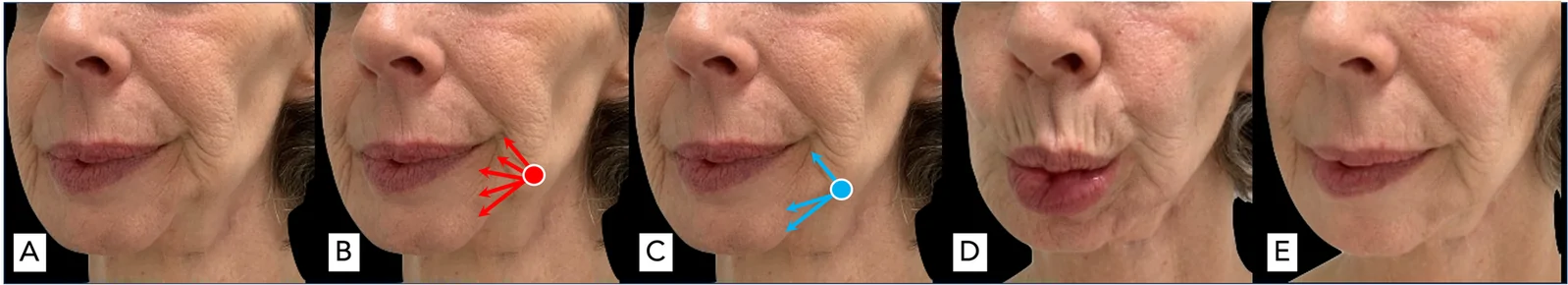
Combined regenerative and volumizing treatment of a 61-year-old female patient with a history of left mandibular osteosarcoma reconstructed with a fibular free flap after a total of five surgeries. (**A**) Baseline appearance demonstrating soft tissue deficiency, contour irregularity, and volume loss affecting the left perioral and mandibular region. (**B**) Initial regenerative treatment using 2 ml of PN administered subdermally. Subcision followed by fan-shaped retrograde linear threading was performed from a single entry point (red circle) to improve tissue quality and release fibrotic adhesions within the reconstructed area. (**C**) Structural restoration using 1 ml of highly cross-linked HA (Saypha Volume Plus) delivered through the same entry point (blue circle). The filler was placed by fan-shaped retrograde linear threading within the marionette region and along the mandibular contour to restore soft tissue volume, improve contour continuity, and enhance facial symmetry. (**D**) Immediate post-treatment result in the dynamic face under lip pouting demonstrating improved contour definition and soft tissue support. (**E**) Immediate post-treatment result in the static face demonstrating improved contour definition and soft tissue support

In a 61-year old female patient with left perioral and mandibular soft tissue deficiency, contour irregularity and volume loss following reconstruction of a left mandibular osteosarcoma with a fibular free flap (five operations in total, no radiotherapy), a single cannula-based session combined PN (2 ml Polyphil) along the mandibular border with selective release of the underlying fibrotic adhesions, followed by volume restoration using a highly cross-linked HA (1 ml Saypha Volume Plus) placed within the marionette line region and along the mandibular contour. Immediately after treatment, symmetry and contour appeared improved in both the static and dynamic face (Fig. 3; Table 2).

Collectively, evidence in postsurgical oncological patients remains largely observational, highlighting both the novelty of the approach and the need for prospective evaluation. Nevertheless, the functional improvements observed in these selected cases, the favourable reported safety profile of PN, and the reversible nature of HA support further investigation of a regeneration-first add-on as a potentially low-morbidity strategy. Because PN, subcision and HA were combined in these cases, the contribution of each component cannot be determined. Future studies should therefore focus on functional, communicative and quality-of-life outcomes in this patient population.

### Congenital malformations

The principal congenital indication in oral and maxillofacial surgery is the secondary deformity of the repaired cleft lip. Even after technically successful primary repair, patients are commonly left with philtral scarring and scar pull, vermilion notching, asymmetry of the cupid’s bow, upper lip volume deficiency and, in some cases, lip incompetence. These features are often persistent, highly visible and present from childhood, so their correction has a substantial effect on self-image and social confidence into adult life.

Surgery remains the mainstay for major secondary deformity, but it is not suitable for every patient and does not address minor asymmetry and volume deficiency well. A minimally-invasive approach is supported by a growing literature. Schweiger et al. reported the first use of injectable HA to correct cleft-lip asymmetry [46], and Rivers et al. subsequently described a dedicated adult cleft facial augmentation clinic in which twenty patients received HA to the upper lip, most often for volume but also for vermilion notch and cupid’s bow asymmetry [47]. Thomson et al. captured the patient’s own perspective with the cleft-specific CLEFT-Q instrument and recorded a statistically significant improvement in self-perceived lip appearance with a low complication profile [48]. BoNT-A is relevant here beyond its dynamic effect: the double-blind randomized vehicle-controlled trial of Chang et al. showed that injection into the orbicularis oris muscle immediately after cleft-lip scar revision improved the resulting scar by reducing tension during healing, which provides a direct rationale for combining tension reduction with scar conditioning and contouring [62]. What this literature does not yet describe, and what the present approach proposes, is the deliberate sequencing of PN conditioning before reversible contouring in this group.

**Fig. 4:**
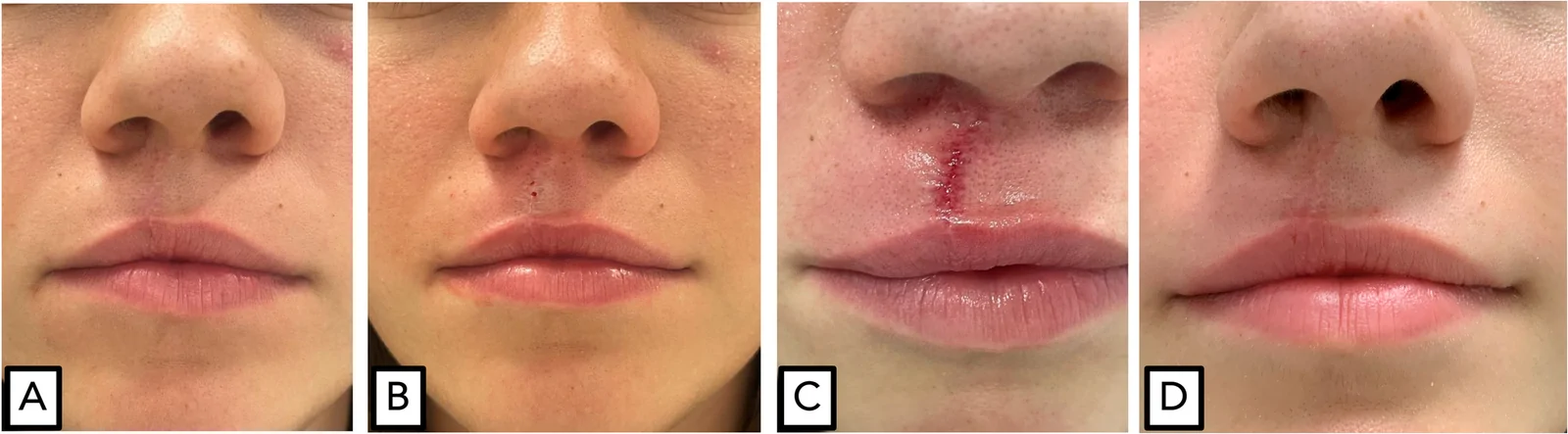
Staged regenerative treatment of secondary cleft lip deformity in a 17-year-old patient using PN (1 ml PolyPhil per session). (**A**) Baseline appearance demonstrating philtral scarring, contour irregularity and vermilion asymmetry following primary cleft lip repair. (**B**) Immediately after the first PN treatment, administered with a needle to condition the philtral scar and improve tissue quality and pliability. (**C**) Immediately after the second PN treatment, performed with a sharp needle in combination with needling (**D**) Four weeks after the second treatment, demonstrating sustained scar softening with a more natural and symmetric vermilion contour

A representative case from the consultation makes the principle concrete. A 17-year-old patient after cleft-lip repair presented for planned lip build-up and scar treatment, with philtral scarring and scar pull, irregular contour, vermilion asymmetry, structural and volume deficits and incipient functional limitation. The stated goals were symmetry and a natural contour, and the treatment was deliberately staged. The philtral scar was first conditioned with PN (1 ml PolyPhil per session), delivered with a sharp needle and, at the second session, in combination with needling; only then was a cannula used to release the residual tethering, with right-sided augmentation to restore symmetry. The sequence was associated with progressive softening of the philtral scar followed by a more natural and symmetric vermilion. (Fig. 4) The case illustrates the conditioning-then-contouring logic: volume is added only after the scar has become pliable enough to move. The reversibility of HA is a particular advantage in this young, lifelong patient group, in whom a permanent material would be undesirable.

The current evidence base for the non-surgical management of secondary cleft deformity is composed mainly of the small series, case reports and patient-reported outcome studies [46–48] rather than controlled trials. While HA constitutes only a temporary mode of volume replenishment, it is nonetheless a repeatable, reversible and well-tolerated option that can defer or complement surgery, and the combination with tension-reducing BoNT-A at the time of any scar revision is a logical and evidence-supported refinement of established practice.

### Trauma and scars

Facial trauma and elective osteotomy including fractures, soft-tissue injuries, gunshot wounds and procedures such as genioplasty, can leave scars that are contracted, adherent or hyperdynamic. The characteristic findings are restricted mobility, mimetic imbalance and aesthetic deficits, united by a single functional principle: tissue that does not glide freely, as anatomically intended, does not move naturally.

This is the category with the strongest controlled evidence for an injectable adjunct. BoNT-A injected adjacent to a healing wound reduces the muscular tension that distracts the wound edges and modulates fibroblast differentiation. PN can add a regenerative component: Belmontesi reported the first use of PDRN for a disabling hypertrophic retracting scar [63], and the multicentre series of Kim et al. described accelerated resolution of post-traumatic, post-surgical and burn scars, including combat injuries, frequently in combination with platelet-rich plasma or BoNT-A [26]. The underlying A2A-mediated anti-inflammatory and pro-angiogenic mechanism is the same one that supports the use of PN in acne and post-surgical scars [38]. From this biology and our clinical experience, we derived the following algorithm, which has not yet been tested in controlled studies: subcision releases the tether, PN condition the released tissue, HA corrects any residual volume deficit, and BoNT-A rebalances hyperdynamic pull across the scar, applied as a single staged concept rather than as isolated treatments.

**Fig. 5:**
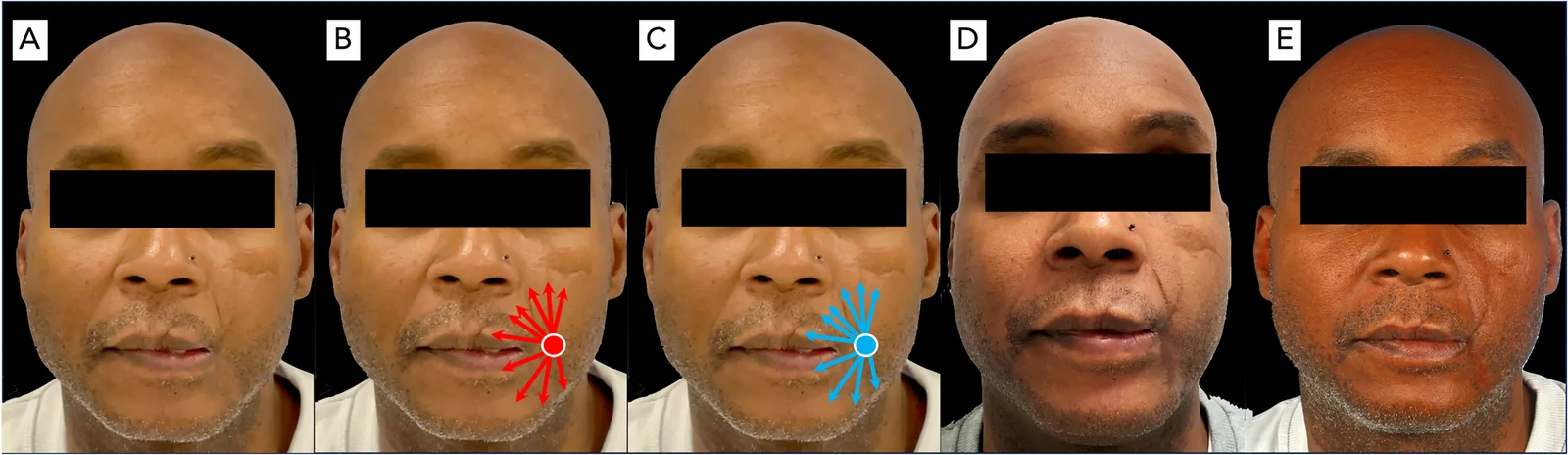
Multimodal treatment of post-traumatic facial scarring and soft tissue tethering in a 44-year-old male patient following a bicycle accident, with multiple prior operations up to the age of nine and a most recent upper-lip scar revision two years earlier. (**A**) Baseline appearance demonstrating scar-related soft tissue retraction, contour irregularity, and tethering affecting the perioral region. (**B**) Initial regenerative treatment with 2 ml of PN (red) (Polyphil), delivered through fan-shaped retrograde linear threading from a single entry point to promote tissue remodeling and release scar-associated adhesions. (**C**) Subsequent structural restoration with 1 ml of medium cross-linked HA (blue) (Saypha Filler) administered through the same entry point to restore soft tissue support, improve contour continuity, and enhance tissue mobility. (**D**) Clinical appearance two months after the treatment, demonstrating improved soft tissue integration, reduced scar-related retraction and restoration of dynamic perioral contour. (**E**) Clinical appearance four months after the treatment, demonstrating improved soft tissue integration, reduced scar-related retraction and restoration of dynamic perioral contour

**Fig. 6:**
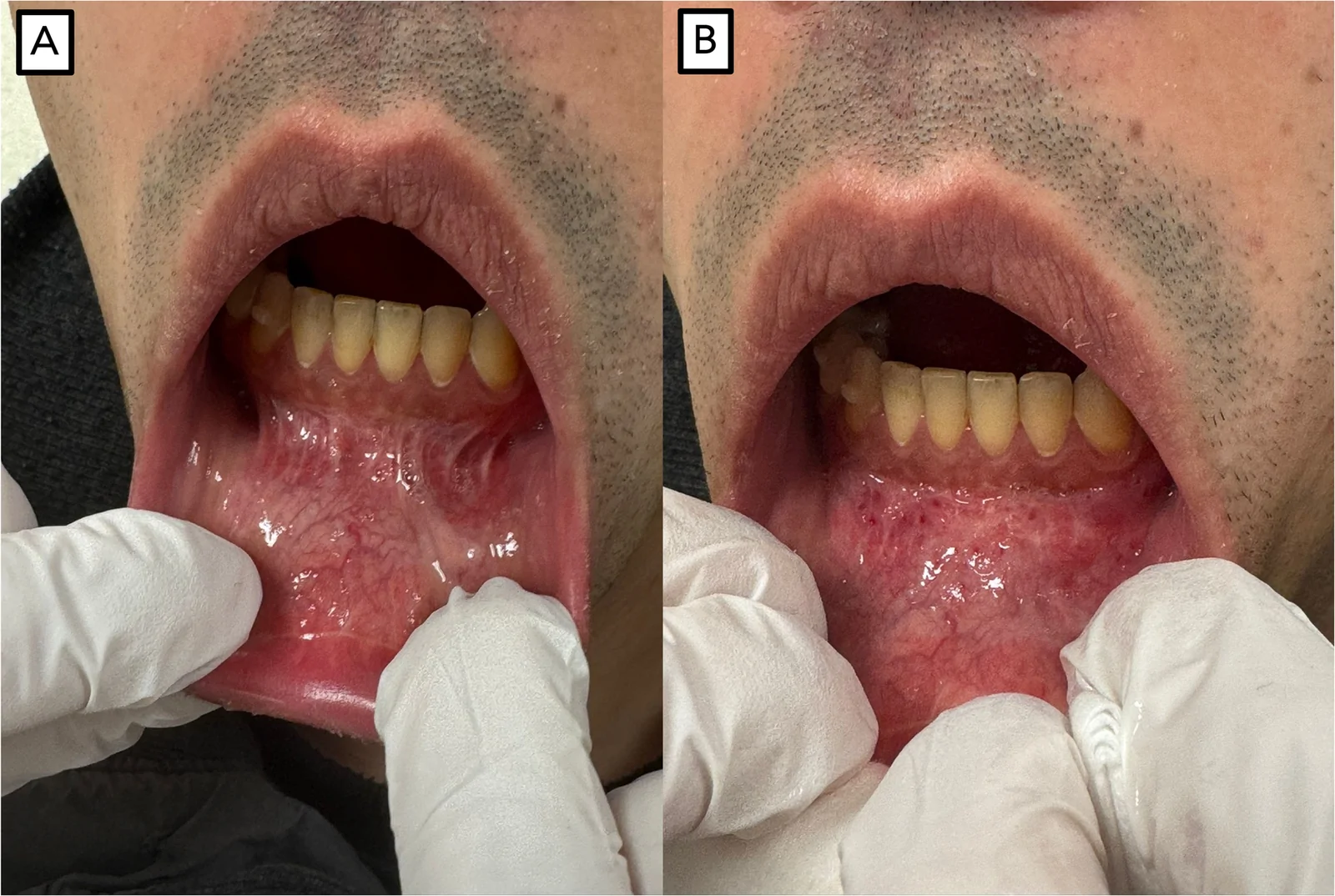
Intraoral release of a symptomatic scar contracture of a 42-year-old patient following genioplasty, treated with PN and needle-subcision technique. (**A**) Baseline appearance demonstrating scar tethering after genioplasty, causing a pulling sensation on lower-lip movement with restricted mobility and discomfort. (**B**) Four weeks after intraoral treatment with 2 ml of PN (Croma-Pharma, Leobendorf, Austria), injected both deep and superficially with a 30G needle in combination with needle subcision, showing released scar tethering, improved lower-lip mobility and resolution of the tethering sensation during animation, with smoother, more symmetric lower-lip excursion on movement

Two cases illustrate the importance of sequence and how the staged approach can be applied at both intra-oral and extra-oral sites. In the first, a 44-year-old male patient presented with contracted scarring and a disturbed perioral aesthetic appearance after a bicycle accident. He had undergone multiple prior operations up to the age of nine, most recently an upper-lip scar revision two years earlier. Treatment was staged through a single entry point: regenerative conditioning with PN (2 ml Polyphil) to release the scar-associated adhesions, followed at the second session by structural restoration with medium cross-linked HA (1 ml Saypha Filler). At four months, reduced scar-related retraction and an improved perioral contour were observed without further open surgery. *(*Fig. 5*)* The second case demonstrates that this work can be targeted and intra-oral: In a 42-year old patient, a symptomatic scar contracture after genioplasty, causing a tethering sensation on lower-lip movement with restricted mobility and discomfort, was released mechanically through needle-subcision with subsequent injection of PN (2 ml Polyphil), placed both deep and superficially with a 30G needle, thereby treating the tether at its source rather than masking it from the skin surface. Four weeks later the tethering sensation had resolved, with improved lower-lip mobility and a smoother, more symmetric lower-lip excursion on movement. *(*Fig. 6*)*

Because the scar-prevention evidence for BoNT-A is now supported by multiple randomized trials and meta-analyses, the strongest practical recommendation in this category is to consider peri-incisional BoNT-A at the time of any planned scar revision, complemented by PN conditioning, selective subcision and HA as the regenerative and structural elements. Controlled data combining these modalities specifically in the maxillofacial trauma patient are still lacking, and represent an attractive target for the prospective study that the foregoing single-agent trials make both feasible and overdue.

### Aesthetic patients

In the maxillofacial setting the aesthetic patient is rarely the cosmetic walk-in. More often this category comprises patients with functional-aesthetic sequelae after excisional or reconstructive surgery, and survivors for whom appearance is an integral part of rehabilitation rather than an elective enhancement. The justification is the same body-image evidence cited at the outset: the prevalence data of Fingeret et al. and the distress estimates of Macias et al. mean that when a large majority of head and neck patients report appearance concerns and a substantial minority experience clinically significant distress, restoring harmony and dynamic balance is a legitimate rehabilitative goal rather than a vanity service [8, 10]. 

**Fig. 7:**
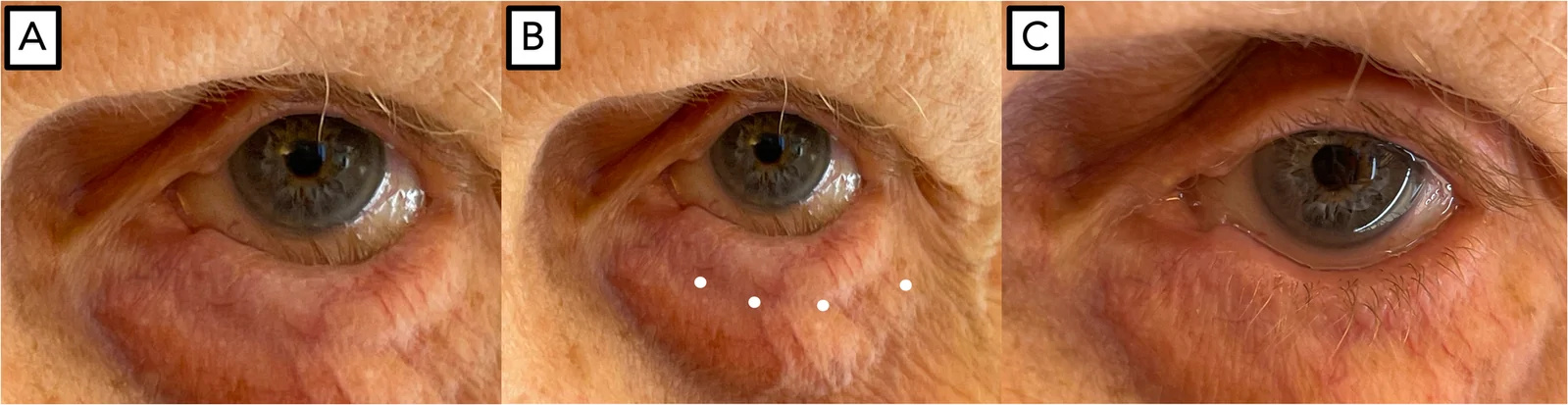
Entropion correction in an 81-year-old male patient following basal cell carcinoma excision on the lower eyelid. The patient reported of an itching sensation and watering to the left eye. (**A**) Baseline appearance demonstrating lower-lid malposition of entropion type with reflex epiphora. (**B**) Injection scheme: BoNT-A (letibotulinumtoxinA, Letybo, Croma Pharma, Leobendorf, Austria) administered at 1 IU per point at four equidistant points approximately 1 cm below the lower lid margin, aligning roughly with the inferior border of the tarsal plate. (**C**) Post-treatment result after two weeks demonstrating corrected lid dynamics and resolution of the reflex epiphora, stable at one-year follow-up with no further treatment required

One clear example from the consultation is an 81-year-old male patient seen after excision of a periocular basal-cell carcinoma, who reported an itching sensation, a watering eye and presented with a lower-lid malposition of entropion type. A single BoNT-A treatment (letibotulinumtoxinA, Letybo), administered at 1 IU per point at four equidistant points approximately 1 cm below the lower lid margin, corrected the lid dynamics and the reflex epiphora, with resolution already evident at two weeks and the result remaining stable at one year with no further treatment required. (Fig. 7) This case underlines two messages at once: (i) that a correctly targeted, low-morbidity intervention can resolve a genuinely functional problem the excision had left behind, and (ii) that the benefit may outlast the expected pharmacological duration of BoNT-A, although spontaneous improvement cannot be excluded in a single case.

This category also defines the ethical boundary of the consultation. The aim is harmony, proportion, symmetry, naturalness and function, not cosmetic escalation, and a clear caution applies to the over-treated face [64, 65]: where the history of previous injectable treatment cannot be established, further injection is withheld. The reversibility of HA and the self-limiting action of BoNT-A support a conservative, reversible-by-default philosophy that is particularly appropriate in patients who have already undergone extensive surgery.

## Discussion

The central proposition of this overview is that aesthetic and regenerative medicine may function as a low-morbidity functional extension of surgical reconstructive pathways in oral and maxillofacial surgery rather than as a cosmetic supplement. The postsurgical face frequently remains limited by impaired tissue quality and disturbed dynamic function long after anatomical continuity has been achieved, a burden that conventional revision surgery, with its added morbidity and need for general anaesthesia, is poorly suited to relieve. The argument advanced here is that a multimodal injectable algorithm may address precisely these residual deficits in an outpatient setting, and that its coherence rests less on the individual agents than on the order in which they are applied: mechanism dictates sequence. In our experience, achieving sustained results in the functional and aesthetic realms typically requires several treatment sessions, with the individual number heavily depending on the severity of clinical findings. This approach is characterized by practical advantages associated with its low-morbidity character: minimal downtime, no need for additional surgery and short treatment times.

The residual scar after reconstruction is, in essence, a problem of persistent low-grade inflammation and disordered collagen, often compounded by radiation-induced fibrosis and hypovascularity. PN act on that environment through the adenosine A2A receptor, damping NF-κB-driven inflammation, supporting fibroblasts and promoting angiogenesis, which provides the biological rationale for a regenerative conditioning step before volume restoration. The sequence is not merely procedural but biomechanical: a fibrotic, poorly perfused bed resists predictable product placement, integrates filler poorly, and concentrates mechanical stress at the very interface where vascular compromise is most likely, so conditioning the tissue first may also have a safety benefit, although this has not been demonstrated clinically [66]. BoNT-A contributes by reducing wound tension and modulating the fibroblast behaviour that drives unfavourable scarring, and by rebalancing dynamic asymmetry through selective muscle weakening, a degree of graded, reversible control over facial movement that static surgery cannot offer. In the pre-treated bed, HA then supplies controlled, reversible volume where it is genuinely required. The fat-grafting work of Rigotti et al. supports a related regenerative principle at the tissue level and supports the wider argument that radiation damage can be partly reversed rather than only camouflaged [67]. The novelty of the present model is not any single agent but the explicit ordering of these established mechanisms into one staged, multimodal, patient-category-specific pathway.

The approach may extend the reach of reconstruction into domains that surgery addresses poorly and widen the therapeutic window for patients who are poor candidates for revision surgery, including the irradiated, the comorbid and the elderly [68, 69]. 

This patient cohort also reframes how success should be measured. The outcomes that matter here such as oral competence, lid function, dynamic symmetry and quality of life are not captured by the wrinkle-severity and global-aesthetic-improvement scales that dominate the cosmetic literature, and a treatment judged ineffective on a wrinkle score may be clinically meaningful for a patient who can again drink without spillage. Future controlled trials should therefore adopt validated functional, scar and patient-reported instruments together with standardized photography and independent raters, and should extend beyond static endpoints to assess emotional expressivity, expression flexibility and interpersonal perception, since restoration of facial dynamics may influence communication in ways that a still photograph cannot register [13, 14]. 

The evidence landscape for the treatments included in our regimen should be stated honestly. It is strongest for BoNT-A, where peri-incisional use for scar prevention is supported by randomized controlled trials and meta-analyses [70, 71]. It is growing for PN and PDRN, with randomized data in acne scars and post-thyroidectomy scars [36, 38] as well as systematic-review support in periodontal and mucosal regeneration [40] although most facial-scar evidence remains observational, as in the case report of Belmontesi [63]. While HA shows a strong evidence base for purely aesthetic indications such as midfacial volumization [72] and lip contouring [73], literature on its use in post-surgical facial rehabilitation is scarce and covers largely small series and case reports [47, 74]. Controlled data specific to the postsurgical maxillofacial patient remain limited across all modalities, and the oncologic-safety discussion that surrounds regenerative tissue transfer, framed by Correas et al. in their recent scoping review [75], should keep clinicians vigilant when regenerative agents are used in oncological patients, even though PN have a favourable reported safety profile to date. Crucially, the evidence that does exist is almost entirely single-agent: the combinatorial, sequenced use that defines the present approach has not itself been tested against its individual components, and it remains formally unknown whether staging adds benefit beyond the sum of the parts. No consensus recommendations for multimodal regenerative treatment plans currently exist for maxillofacial patients, and generating them will require trials designed around the sequence itself rather than around any one agent. In the present cases, this limitation is compounded by the frequent combination of subcision, PN and HA within one session and by the absence of validated outcome measures and controls; the clinical observations reported here are therefore hypothesis-generating only.

Safety is the precondition for everything above, and it is principally a matter of anatomy. The rare but largely irreversible vascular and visual complications of injectable treatment concentrate in the central and upper midface and are, to a considerable extent, preventable. As the danger-zone and filler-complication literature summarized by Beleznay et al. [44, 45, 76] makes clear, knowledge of the vascular danger zones, discipline regarding injection plane, a vessel-aware technique and immediate availability of a complication pathway substantially reduce this risk [76]. In our consultation, suspected vascular occlusion is managed according to published protocols: injection is stopped immediately, high-dose hyaluronidase is infiltrated throughout the affected vascular territory and repeated until reperfusion is noted. In case of visual symptoms, urgent ophthalmological referral is initiated. Even with prompt treatment, visual loss is frequently irreversible, so the reversibility of HA does not guarantee recovery of every instance of ischemia and subsequent potential necrosis. In previously operated and irradiated tissue, vascular anatomy may additionally be altered, which is a further reason for blunt cannulas and small volumes. Embedding the consultation within an oral and maxillofacial surgery department, where this anatomical expertise and the necessary emergency infrastructure already exist, is therefore not only convenient but is itself a safety argument for the model.

## Conclusion

Modern maxillofacial reconstruction increasingly extends beyond anatomical continuity. Across tumor, congenital, traumatic and aesthetic cases, deficits in tissue quality, scar mobility, dynamic balance and facial function often persist after successful surgery. Minimally-invasive regenerative and aesthetic treatment may serve as a biologically guided extension of reconstructive rehabilitation, with PN, HA and BoNT-A offering complementary capabilities: regenerative tissue conditioning, reversible structural restoration and selective dynamic modulation. Applied within a staged, mechanism-led framework, they may improve oral competence, tissue gliding, dynamic symmetry and quality of life with low morbidity and without renewed open surgery. The central concept is restoration of tissue behaviour and function, where small, targeted interventions may yield meaningful benefit in patients inadequately served by revision surgery alone. Given the illustrative, non-controlled nature of the cases presented, these observations are hypothesis-generating and require confirmation in prospective studies with validated functional, scar and patient-reported outcome measures.

## Data Availability

No datasets were generated or analysed during the current study.

## Declarations

### AI use

During the preparation of this work, AI-assisted tools were used in part to support language editing and translation. The authors subsequently reviewed and revised the content as appropriate and take full responsibility for the final content of the publication.
